# Clinical Characteristics and Outcomes for Neonates with Respiratory Failure Referred for Extracorporeal Membrane Oxygenator (ECMO) Support

**DOI:** 10.3390/children12070925

**Published:** 2025-07-13

**Authors:** Pooja Musuku, Keith Meyer, Felipe E. Pedroso, Fuad Alkhoury, Balagangadhar R. Totapally

**Affiliations:** 1 Division of Critical Care Medicine, Nicklaus Children’s Hospital, Miami, FL 33155, USAbalagangadhar.totapally@nicklaushealth.org (B.R.T.); 2 Herbert Wertheim College of Medicine, Florida International University, Miami, FL 33155, USA; 3 Department of Critical Care Pediatric Surgery, Nicklaus Children’s Hospital, Miami, FL 33155, USA

**Keywords:** ECMO, neonatal respiratory failure, oxygenation index, vasoactive–inotropic score, persistent pulmonary hypertension of the newborn

## Abstract

**Objective**: The aim of this study was to describe the presenting characteristics and outcomes of neonates with respiratory failure referred for extracorporeal membrane oxygenation (ECMO) support, compare those who received ECMO support (ECMO group) to those who did not (non-ECMO group), and evaluate the predictive variables requiring ECMO support. **Methods**: All neonates (<15 days) with respiratory failure (without congenital diaphragmatic hernia or congenital heart disease) referred to our regional ECMO center from 2014 to 2023 were included in this retrospective study. Patient demographics, birth history, and clinical and outcome variables were analyzed. Oxygenation indices and vasoactive–inotropic scores obtained at PICU arrival and four hours after arrival were compared between the two groups using ROC analysis, with ECMO initiation as an outcome variable. Youden’s index was used for optimal threshold values. Chi-square, Mann–Whitney U, and binary logistic regression were used for comparative analyses. **Results**: Out of the 147 neonates, 96 (65%) required ECMO support. The two groups significantly differed in the prevalence of pulmonary hypertension (pHTN; systemic or suprasystemic pulmonary pressures), lactate level, and oxygenation indices. Mortality was not different between the two groups. Presence of oxygen saturation index (OSI) ≥ 10 had a sensitivity 96.8% in predicting the need for ECMO support. On regression analysis, OSI and pHTN were independent predictors of ECMO support. **Conclusions**: Oxygenation indices and echo findings predict the need for ECMO support in neonatal hypoxemic respiratory failure. These findings help non-ECMO centers make appropriate and timely transfers of neonates with respiratory failure to ECMO centers.

## 1. Introduction

Meconium aspiration syndrome (MAS), persistent pulmonary hypertension of the newborn (PPHN), and congenital diaphragmatic hernia (CDH) account for 75% of all neonatal respiratory indications for extracorporeal membrane oxygenation (ECMO) support [1]. Although the total number of ECMO cases across all age groups increased annually, the proportion of neonatal ECMO cases declined—from 49.7% of all ECMO cases in 2001 to 25.7% in 2010, and further from 21.2% in 2011 to just 8.1% in 2016 [1]. The decrease in the relative frequency of neonatal ECMO is related to both an actual decrease in ECMO support in neonates and an increase in ECMO use in other age groups [1,2]. This can be attributed to advances in medical management, including high-frequency oscillatory ventilation (HFOV), exogenous surfactant therapy, and inhaled nitric oxide (iNO), which have also reduced the need for ECMO support, shortened the duration of hospitalization, and decreased mortality for these patients [3,4].

Recent data indicate a decline in survival to hospital discharge for neonates receiving ECMO for respiratory failure—while the overall survival rate since 1990 is 72%, it dropped to 67% during the 2012–2017 period [1]. This trend may be explained by broader inclusion criteria, which have allowed ECMO to be used for a wider range of critically ill patients, including those with comorbidities who were previously not considered candidates [1].

Significant variability exists in the selection criteria for neonatal ECMO [5]. The 2017 Extracorporeal Life Support Organization (ELSO) criteria for neonatal ECMO suggest considering ECMO with an oxygenation index cutoff of more than 40 for 4 or more hours [6]. However, a majority of neonates who receive ECMO are outborn, and the optimal criteria for referral of a sick neonate to an ECMO center remain unclear [7,8]. Survival has been reported to be better (77%) with early referrals within the first 24 h of life, compared to late referrals (54%) [9].

This study’s objectives were to describe the clinical characteristics and outcomes of neonates with respiratory failure referred for ECMO support to our institution, compare the differences between the neonates who received ECMO support (ECMO group) and those who did not receive ECMO (non-ECMO group), and to evaluate the predictive variables for ECMO support.

## 2. Materials and Methods

### 2.1. Study Design

This single-center, retrospective cohort study analyzed patients transferred to a regional ECMO program at Nicklaus Children’s Hospital (NCH), Miami, FL, USA during 2014–2023. The ECMO support for neonatal respiratory failure at NCH is provided in the pediatric intensive care unit (PICU). Neonates are transported to the NCH ECMO center from NICUs in the region or transferred from the in-house level IV NICU. Our institutional guidelines suggest transferring a neonate with an oxygenation index (OI) of 25 or more to the PICU for ECMO watch, defined as a period of intensive monitoring and ongoing assessment for potential ECMO candidacy. ECMO initiation is typically considered when the OI is 40 or more, or in the presence of severe hemodynamic instability. The data was retrieved from patients’ medical charts and the VPSLLC (Virtual Pediatric Systems) database for hospital patients.

**Figure 1:**
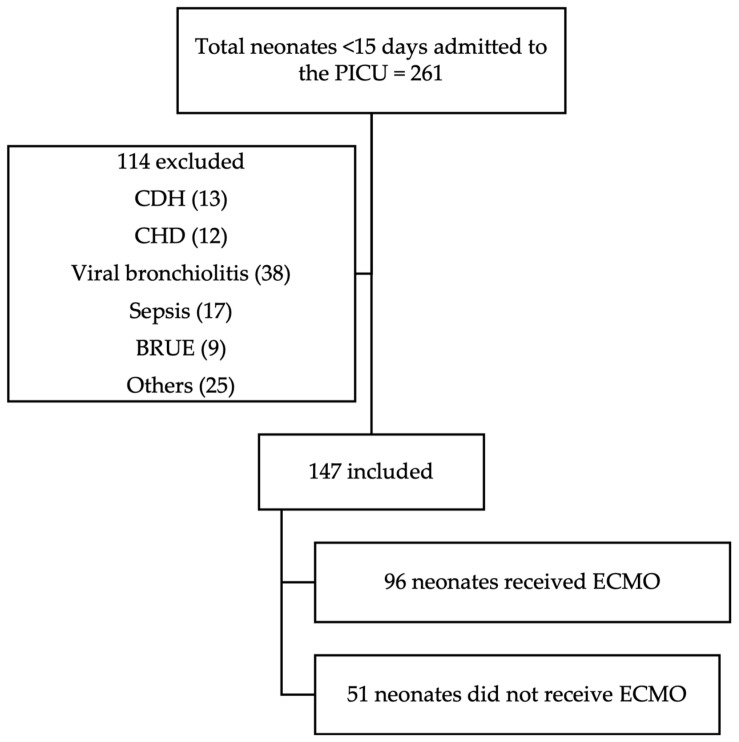
CONSORT diagram. Abbreviations: CDH—congenital diaphragmatic hernia; CHD—congenital heart disease; BRUE—brief resolved unresponsive event; others: hyperammonemia, hypocalcemia, intestinal surgery, non-accidental or accidental trauma, and arteriovenous malformations.

All neonatal patients (<15 days old) admitted to the NCH PICU during the study period were reviewed for potential inclusion. Of the 261 neonates admitted, 147 met the inclusion criteria and were included in the final analysis. Inclusion criteria consisted of age <15 days and admission to the PICU specifically for ECMO watch. Neonates with congenital diaphragmatic hernia or congenital heart disease were excluded, as were those admitted to the PICU for diagnoses unrelated to ECMO (Figure 1).

### 2.2. Data

Demographic data (age, sex, race, and ethnicity), perinatal details (gestational age, birth weight, mode of delivery, APGAR scores at 1, 5, and 10 min), and relevant maternal history were retrieved. Other relevant events collected included cardiopulmonary resuscitation (CPR) in the delivery room or pneumothorax before arrival at the ECMO center.

Laboratory values, including hemoglobin and platelet count, were collected upon arrival at the PICU. Lactate levels and fluid boluses documented within the first 4 h of PICU admission or before ECMO initiation, whichever was earlier, were used for analysis. Echocardiogram data (available in 145/147 patients) closest to PICU admission, whether from an outside hospital or after arrival at NCH, were used to determine the presence of pulmonary hypertension (pHTN). PHTN was diagnosed if there was a demonstration of systemic or more than systemic pulmonary pressures such as right to left or bidirectional shunting at the PDA level, or tricuspid regurgitation jet of more than or equal to half systemic pressures or actual documentation of pulmonary pressures on the echocardiogram report.

Clinical information, such as blood gases, oxygenation indices, vasoactive–inotropic scores (VIS), and the use of systemic pulmonary vasodilators other than nitric oxide (such as milrinone or iloprost), was gathered at two specific time points: A and B. The data acquired upon departure from the referring institution or arrival at the PICU was labeled Time A. The data collected four hours after arrival at the PICU or before initiation of ECMO support, whichever was the earliest, was labeled Time B. We chose the 4 h mark from the time of PICU arrival for Time B values as the median time for placement on ECMO was around 3 h.

Oxygen saturation index (OSI) and saturation/FiO_2_ (SF) ratios were collected for all patients, and OI, PaO_2_/FiO_2_ (PF) ratio and A-a gradient were collected when arterial blood samples were available (for 119 patients at Time A and 123 patients at Time B, out of 147 patients total). The following formulas were used for the calculation of oxygenation indices:

- Alveolar–arterial (A-a) oxygen gradient: Alveolar PO_2_ (PAO_2_)- arterial PO_2_ (PaO_2_).
- SF ratio: SaO_2_/FiO_2_ (calculated using preductal oxygen saturation) [10].
- PF ratio: PaO_2_/FiO_2_ (calculated using arterial blood sample PaO_2_) [10].
- Oxygen saturation index (OSI): (FiO_2_ × mean airway pressure) divided by SaO_2_ [10,11].
- Oxygenation index (OI): (FiO_2_ × mean airway pressure) divided by PaO_2_ [10,11].
- Vasoactive–inotropic score (VIS): dopamine dose (μg/kg/min) + dobutamine dose (μg/kg/min) + 100 × epinephrine dose (μg/kg/min) + 10 × milrinone dose (μg/kg/min) + 10,000 × vasopressin dose (U/kg/min) + 100 × norepinephrine dose (μg/kg/min) [12].

For neonates requiring ECMO support, cannulation time and total duration of ECMO support (hours) were documented. Peripheral venoarterial (VA) cannulation was consistently used in all patients in the cohort. Reasons for decannulation were noted as routine due to improvement of primary condition, ECMO complications, death on ECMO, or removal of ECMO in end-stage conditions requiring withdrawal of life support.

Primary diagnoses included MAS, respiratory distress syndrome (RDS), PPHN, and others (including hemophagocytic lymphohistiocytosis and sepsis). Outcome variables comprised PICU length of stay, hospital length of stay, duration of invasive mechanical ventilation, mortality, and morbidity at discharge. Morbidity was defined as needing a gastrostomy tube, tracheostomy tube, oxygen support, or anti-epileptic medications at discharge.

### 2.3. Data Analysis

Microsoft Excel version 16.87 (Microsoft Corporation, Redmond, WA, USA) was used for data collection. Data was exported and analyzed using SPSS version 29.0 (IBM Corporation, Armonk, NY, USA). Descriptive statistics are presented as percentages for categorical data and medians (interquartile ranges) for continuous data. Demographic, clinical characteristics, and outcome variables were compared between ECMO and non-ECMO groups. Chi-square and Mann–Whitney U tests were used for univariate analysis. Binary logistic regression analysis was used to predict ECMO requirements. The linearity of the continuous variables with respect to the logit of the dependent variable was assessed via the Box–Tidwell (1962) procedure [13]. We generated receiver operating curves (ROCs) for oxygenation indices at Time A and B and obtained optimal cutoff values using Youden’s index to perform sensitivity analysis. The missing values were not imputed, and cases with missing data were excluded from the analyses of the affected variables. A *p*-value ≤ 0.05 was considered statistically significant. We used the STROBE guidelines for reporting observational studies. IRB approved this study as exempt from full review.

## 3. Results

The median gestational age and birth weight of 147 neonates in this study were 39.1 (40.4–37.7) weeks and 3.32 (3.7–3.0) kg, respectively. ECMO support was required in 96 (65%) neonates. ELSO criteria for neonatal ECMO were met at time A in 92 out of 96 neonates in the ECMO group and 42 out of 51 in the non-ECMO group (*p* = 0.012). Demographic and baseline clinical characteristics, including birthing details, ventilator details, vasoactive, pulmonary vasodilator use, and the need for CPR in the delivery room, were similar in both groups (Table 1). The presence of pneumothorax on arrival at the ECMO center and the surfactant administration prior to transfer were similar in both groups. The prevalence of systemic or suprasystemic pulmonary pressure was higher in the ECMO group (80.2% vs. 58.8%; OR: 2.84 (95% CI: 1.34–6.01); *p* = 0.006). All patients except one received nitric oxide before arrival, and all were on nitric oxide at Time B.

Table 2 shows continuous variables, including gestational age, birth weight, and oxygenation indices, compared between the ECMO and non-ECMO groups. Gestational age and birth weight were not different between the groups. The neonates in the ECMO group arrived earlier at the PICU (34 (18.3–58.5) h vs. 46 (32–71) h (*p* = 0.044)). Lactate level and oxygenation indices were significantly different in the two groups (Table 2).

ECMO support was initiated at a median postnatal age of 40.5 (23.3–87.2) hours and a median time of 3 (1.25–10.5) hours after arrival at the ECMO center. The duration of ECMO support was 138 (85–240) hours. Out of all ECMO patients, 82.1% were routinely decannulated due to improvement in primary condition; 10.5% had to be decannulated due to a complication (out of these patients, 8 had intracranial bleeds and 2 had intraabdominal bleeds); 1.1% died while on ECMO; 5.3% were decannulated as part of the withdrawal of life support due to poor prognosis.

Length of stay in the PICU and length of mechanical ventilation were significantly higher in the ECMO group (Table 2). The hospital mortality rate was 20.8% in the ECMO group and 11.8% in the non-ECMO group (OR: 1.97 (0.74–5.28); *p* = 0.256). Morbidity rates were also similar in both groups (Table 2).

**Figure 2:**
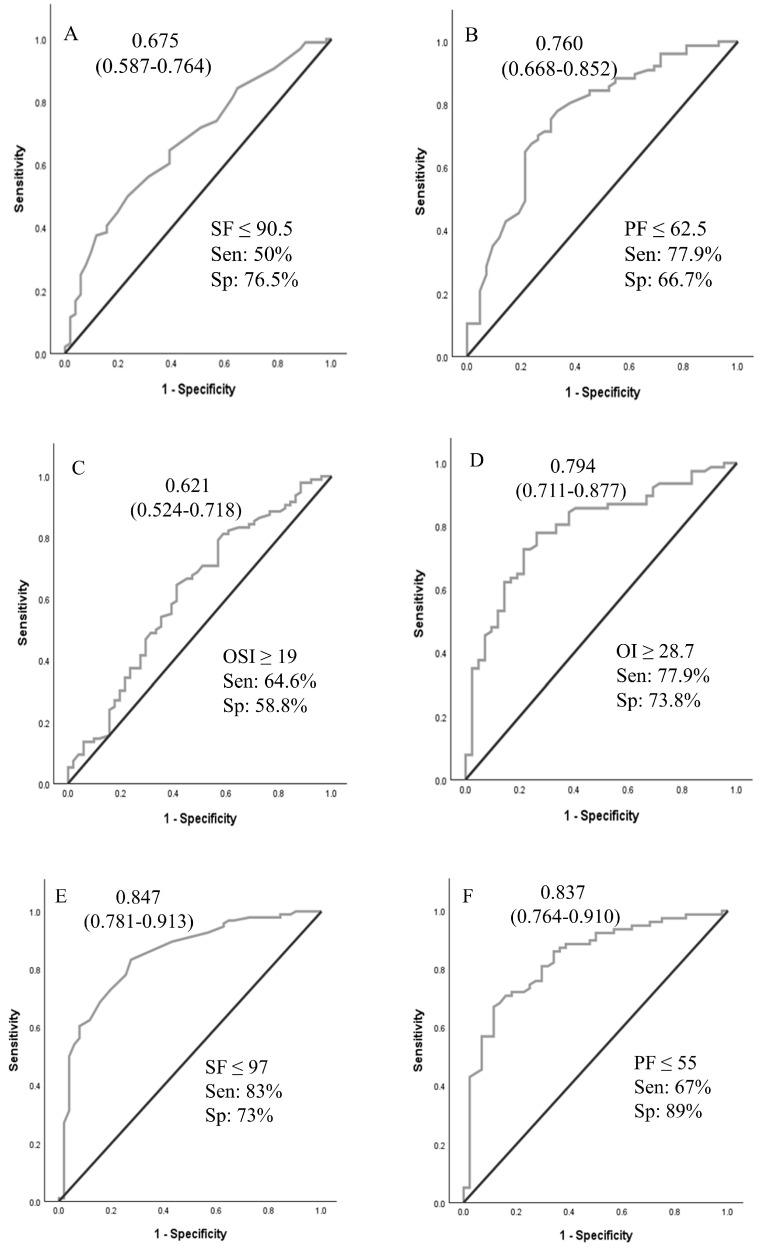

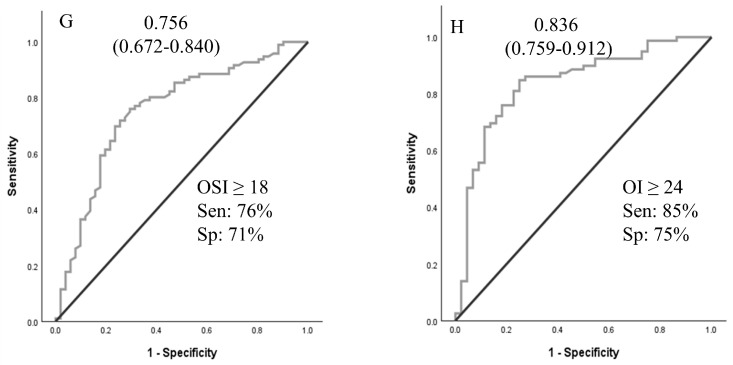
Receiver operating curves for oxygenation indices at time A and time B to predict the need for ECMO support. Area under the ROC curve (95% CI). (**A**–**D**): values at PICU arrival; (**E**–**H**): values at 4 h after PICU arrival. Abbreviations: SF—SpO_2_/FiO_2_ ratio; PF—PaO_2_/FiO_2_ ratio; OSI—oxygen saturation index; OI—oxygenation index.

A logistic regression analysis was performed to ascertain the effects of pHTN, VIS, and OSI at times A and B on the likelihood that neonates received ECMO support. For time A and time B values, pHTN and OSI were independent predictors of ECMO support (Table 3). Using OSI ≥ 10 for ECMO transfer decisions achieved 96.8% sensitivity. ROCs were generated for oxygenation indices (OI, SF, OSI, and PF ratios at times A and B), as shown in Figure 2. ROCs generated at Time B were more accurate than Time A values. The sensitivity analyses and cutoff values are presented in Figure 2.

## 4. Discussion

In our study of neonates with hypoxic respiratory failure who were potential ECMO candidates, oxygenation indices and echocardiographic findings of pHTN demonstrated moderate accuracy in predicting the need for ECMO support. An OI of 28.7 or more at time A predicted the need for ECMO support with 76% accuracy. The presence of oxygen saturation index (OSI) ≥ 10 at time A had a sensitivity of 96.8% for predicting the need for ECMO support. The results of this study may assist neonatal centers in establishing objective criteria for patient transfers to higher levels of care, ensuring transfers are conducted safely and in a timely manner. Implementing such criteria may also help avoid unnecessary transfers, thereby reducing maternal–infant separation and minimizing the healthcare costs associated with inter-facility transport.

RV dysfunction is common in neonates with PPHN due to associated pHTN [14,15]. Fetal and transitional circulation, neonatal respiratory failure and idiopathic pHTN are some of the mechanisms leading to RV dysfunction [15]. Pulmonary hypertension and RV dysfunction were more common in neonates who required ECMO support in our study and severe RV dysfunction is known to be associated with poor outcomes [16].

Overall, there were no significant differences between the infants in the ECMO group and non-ECMO group with respect to demographics, birthing details, ventilator, or vasoactive use. However, oxygenation indices such as OI, OSI, PF, and SF ratios significantly differed between the two groups. Oxygenation indices as measures of severity and risk of mortality in neonates with hypoxic respiratory failure have been well-established [17,18,19].

While the ELSO and individual ECMO centers have established criteria for placing a neonate with hypoxic respiratory failure on ECMO support, clear guidelines for when to transfer to an ECMO center are not well defined. Timely transfer of a neonate who requires ECMO support will ensure clinical stability during cannulation and lead to better outcomes [20]. Gautham and Fernandez recommended initiating transfer to an ECMO center when the OI of 20 or more or A-a gradient of 600 or more [20]. In their opinion, transferring 20 neonates for one to receive ECMO is deemed acceptable. The mortality rate associated with transport must be considered when making such decisions [21]. Boedy et al. recommended transferring neonates with MAS to an ECMO center when they reach an OI of 25 [21]. A-a gradient and OI criteria are used to initiate ECMO support, and the cutoff values were developed based on higher associated mortality. An A-a gradient of 610 torr for 8 h had a sensitivity of 93% and specificity of 71% for mortality related to PPHN [17]. In another similar review of patients, PaO2 < 50 mm Hg for four hours had a sensitivity of 86% and specificity of 96% for mortality [18]. An OI greater than or equal to 40 was associated with a mortality risk of 80–90% in a single-center study [19]. In our analysis, OSI of ≥10 demonstrated high sensitivity for predicting the need for ECMO support. OSI of 10 approximates OI of 18 (OI = OSI × 1.78) [11]. We suggest using an OSI > 10 as a threshold to initiate timely transfer to ECMO centers, or at a lower OSI if there are other associated clinical indicators, such as significant pHTN, hemodynamic instability, or clinical instability.

In our study, an OI of 28.7 at Time A had a sensitivity and specificity for ECMO support of 78% and 74%, respectively. Our cohort had no transport-associated mortality, and the mortality rate was not significantly different between the ECMO and non-ECMO groups. If the findings of our study are confirmed in large multi-institutional data, these criteria can be used to guide the transfer of term neonates to an ECMO center. Additionally, there is a need for local non-ECMO and regional ECMO centers to conjointly develop criteria for when to call the ECMO center and when to begin the transfer process.

A retrospective review from the United Kingdom reported no significant factors (clinical values including oxygenation index or laboratory values) at the time of referral or arrival at the ECMO center that predicted survival [22]. They concluded that the condition of the infant, based on clinical experience, remains the best indicator of the need for transfer [22]. However, in our study, oxygenation indices showed moderate accuracy in predicting the need for ECMO support. Adding the objective criteria of oxygenation indices to the patient’s clinical condition will be helpful for safe and timely transfer.

We have used OSI for regression analysis as there was no missing data. In addition, the OSI is useful for clinical practice as it can be calculated in neonates without an arterial line. An arterial line was not placed or could not be placed in a few neonates in our study. In such situations, the SF ratio or OSI can be used to decide on transfer and ECMO cannulation. The SF and OSI metrics have been validated as indices of the severity of hypoxic respiratory failure in pediatric respiratory failure [10,11,23]. We are presenting the comparative predictive values for SF and OSI, along with arterial blood gas-based oxygenation indices.

There have not been many large-scale comparable studies from the US reviewing referral patterns for neonatal ECMO. A recent report from the UK studied all referrals made for potential ECMO requirements during three years from 2014 to 2017 [24]. Among those referred for ECMO, only 1/3rd of patients eventually required ECMO. Independent risk factors for the need for ECMO were identified as the primary diagnosis (especially CDH), oxygenation index, and vasoactive–inotropic score (VIS). One-fifth of all patients referred for ECMO, irrespective of whether they received ECMO or not, died within 90 days, with a majority of the mortality occurring in the first 24 h of referral [24]. The risk factors we identified in our study for the need for ECMO were systemic/suprasystemic pressures on echo and OSI by regression analysis. In our study, mortality was 17.7% in the total group, with no difference in mortality whether neonates received or not received ECMO support. Among all the patients referred for ECMO in our study, 65% of the neonates were initiated on ECMO, higher than in the UK study. The distance from the receiving hospital, regionalization of healthcare, and the addition of CDH patients in the UK cohort compared to our cohort could have influenced the transfer criteria and eventual need for ECMO support.

In another UK study, out of 469 neonates referred, 288 (61%) patients received ECMO, with a survival rate of 71% [25]. In the same study, reviewing all neonatal deaths during the study period, 110 deaths out of 6905 were noted to be potentially avoidable by ECMO treatment. They noted a significant regional variation in rates of ECMO referral and neonatal deaths, pointing to regional ECMO referral criteria and the care referral plan by the referring NICU as influential factors [25]. The finding of this study further illustrates the need for establishing guidelines for transferring neonates with hypoxemic respiratory failure and timely transfer to an ECMO center.

There are several limitations to this study. This is a single-center retrospective study, and one needs to be careful in generalizing the findings of this study. We excluded CDH patients from our analysis as we had very few patients in the cohort, and their ECMO entry criteria were different than for neonates with hypoxic respiratory failure from other causes [26,27]. This is another limitation for generalizing our findings to all neonates with hypoxic respiratory failure. The extended study period (2014–2023) and advances in clinical practice over time, including evolving criteria for ECMO initiation, may be viewed as limitations. Retrospective studies can be limited by data availability and accurate documentation. In addition, we can only deduce association, not cause-and-effect relationships. The accuracy of the data relied on information from outside hospitals regarding birthing details, which may vary in completeness. Additionally, due to the absence of arterial lines in some cases, accurate oxygenation index (OI) values could not be calculated in all patients, potentially impacting analysis due to missing values. Furthermore, echocardiogram findings are subjective and may vary depending on the interpreting clinician, introducing variability in the assessment of cardiac function.

Despite these limitations, our study contributes valuable insights into clinical predictors for ECMO requirements. Our patient population was unique in the sense that all term neonates who were transferred to our PICU were in potential need of ECMO support. All neonatal transfers who were not potential candidates for ECMO or in whom ECMO support was contraindicated were not transferred to the ECMO center. With no transfer-related mortality and similar mortality in ECMO and non-ECMO groups, the findings of our study are useful for guideline development. Only oxygenation indices and echo findings significantly differed between the two groups as predictors of ECMO support. One of the oxygenation targets (SF, PF, OSI, or OI), along with echocardiographic findings, can be used to establish the criteria for mobilizing ECMO teams and facilitating prompt evaluation and transfers from referring centers. Comparative values of OSI vs. OI and SF vs. PF are helpful for clinical situations without an arterial line.

## 5. Conclusions

Oxygenation indices and echo findings of pHTN predict the need for ECMO support in neonatal hypoxemic respiratory failure. If confirmed in multicenter data, these findings help non-ECMO centers develop guidelines and safely transfer neonates with hypoxic respiratory failure to ECMO centers.

## Figures and Tables

**Table 1:** Demographic and clinical characteristics and outcomes of neonates in ECMO vs. non-ECMO groups.

Variable	ECMO Group ^a^	Non-ECMO Group ^a^	Total Patients ^a^	Odds Ratio (95% CI) *; *p* Value
Male	61 (63.5%)	33 (64.7%)	95 (63.9%)	0.95 (0.47–1.93); 0.889
Race				
White	56 (58.3%)	28 (54.9%)	84 (57.1%)	0.922
Black	12 (12.5%)	7 (13.7%)	19 (12.9%)	
Other	28 (29.2%)	16 (31.4%)	44 (29.9%)	
Ethnicity				
Hispanic	46 (47.9%)	28 (54.9%)	75 (50.7%)	0.559
Not Hispanic	35 (36.5%)	18 (35.3%)	53 (35.8%)	
Unknown	15 (15.6%)	5 (9.8%)	20 (13.5%)	
Delivery type—C/S	67 (69.8%)	37 (72.5%)	104 (70.7%)	0.87 (0.41–1.86); 0.726
CPR in the delivery room	10 (10.4%)	2 (3.9%)	12 (8.2%)	2.849 (0.6–13.53) 0.218 ^b^
Intubation in 24 HOL	87 (90.6%)	45 (88.2%)	132 (89.8%)	0.78 (0.26–2.32); 0.776 ^b^
Surfactant prior to transfer	51 (53.1%)	31 (60.8%)	82 (55.8%)	0.731 (0.37–1.46); 0.373
Transfer type—NICU to PICU	62 (64.6%)	36 (70.6%)	98 (66.7%)	0.76 (0.36–1.58); 0.462
Pneumothorax on arrival	13 (13.5%)	3 (5.9%)	16 (10.9%)	2.506 (0.68–9.24); 0.178 ^b^
Milrinone or iloprost on arrival	39 (40.6%)	17 (33.3%)	56 (38.1%)	1.37 (0.67–2.79); 0.386
HFOV on arrival	76 (79.2%)	36 (70.6%)	112 (76.2%)	1.58 (0.73–3.45); 0.245
HFOV at 4 h	82 (85.4%)	38 (74.5%)	120 (81.6%)	2.0 (0.86–4.68); 0.104
Milrinone or iloprost at 4 h	49 (51.0%)	20 (39.2%)	69 (46.9%)	1.616 (0.81–3.20); 0.171
Fluid bolus in the first 4 h	53 (55.2%)	29 (56.9%)	82 (55.8%)	0.935 (0.47–1.85); 0.848
RV dysfunction (*n* = 145)	23 (24.5%)	5 (10%)	28 (19.4%)	2.92 (1.03–8.22); 0.046 ^b^
PHT	77 (80.2%)	30 (58.8%)	107 (72.8%)	2.84 (1.34–6.01); 0.006
Primary diagnosis				
MAS	58 (60.4%)	27 (52.9%)	85 (57.8%)	0.424
RDS	10 (10.4%)	9 (17.6%)	19 (12.9%)	
PPHN	26 (27.1%)	15 (29.4%)	41 (27.9%)	
Sepsis and others	2 (2.1%)	0	2 (1.4%)	
Outcomes				
PICU LOS (days)	13 (8.3–25)	7 (3–13)	11 (6–22)	<0.001
Total LOS (days)	44 (32.3–72.8)	41 (25–60)	44 (30–65)	0.180
Mechanical ventilation (days)	25 (16–32)	18 (14–27)	23 (15–31)	0.023
Morbidity (*n* = 121)	19 (25%)	9 (20%)	28 (23.1%)	1.33 (0.54–3.27); 0.528
Mortality	20 (20.8%)	6 (11.8%)	26 (17.7%)	1.97 (0.74–5.28); 0.256 ^b^
Morbidity or mortality	39 (40.6%)	15 (29.4%)	54 (36.7%)	1.64 (0.79–3.4); 0.179

* Odds ratios with 95% confidence intervals are presented where applicable for univariate analysis of descriptive variables; for others, only *p* values are presented. ^a^ Categorical variables presented as *n* (%) and continuous variables presented as median (IQR); ^b^ Fisher exact test. Abbreviations: A-a gradient—alveolar–arterial oxygen gradient; ECMO—extracorporeal membrane oxygenation; FiO_2_—fraction of inspired oxygen; HFOV—high-frequency oscillatory ventilation; MAS—meconium aspiration syndrome; morbidity = the need for a gastrostomy tube, tracheostomy tube, oxygen support, or anti-epileptic medications; OI—oxygenation index; OSI—oxygen saturation index; PHT—systemic or suprasystemic pulmonary pressures on echocardiogram; PICU—pediatric intensive care unit; PF ratio—ratio between PaO_2_ and FiO_2_; PPHN—persistent pulmonary hypertension of the newborn; RDS—respiratory distress syndrome; ROM—rupture of membranes; SF—ratio between SpO_2_ and FiO_2_; VIS—vasoactive–inotropic score.

**Table 2:** Comparison of continuous variables between the ECMO and non-ECMO groups.

Variable	ECMO Group	Non-ECMO Group	Total Patients	*p* Value
Age in hours at PICU admission	34 (18.3–58.5)	46 (32–71)	37 (24–66)	0.044
Gestational age	39.3 (38–40.4)	39 (36.9–40.1)	39.1 (37.7–40.4)	0.084
Birth weight in kilograms	3.34 (3–3.6)	3.32 (2.8–3.9)	3.32 (3.0–3.7)	0.478
APGAR at 1 min	7 (4–8)	7 (5–8)	7 (5–8)	0.604
APGAR at 5 min	8 (6–9)	8 (7–8.3)	8 (6–9)	0.907
Maximum lactate recorded in the first 4 h	3.7 (2.2–5.8)	2.3 (1.3–4)	3.3 (1.9–5.1)	<0.001
Fluid boluses received in the first 4 h of admission (mL/kg)	10 (0–20)	10 (0–20)	10 (0–20)	0.776
Values at PICU arrival (Time A)				
A-a gradient (*n* = 119)	607.8 (588.4–625.9)	570.3 (488.3–607.7)	601.3 (567–621.3)	<0.001
SF ratio	90.5 (82.5–96)	94 (91–98)	92 (85–97)	<0.001
PF ratio (*n* = 119)	46 (38–61.5)	68.1 (52.8–112.5)	52 (40–70)	<0.001
OSI	20.1 (17.4–23.7)	18.2 (14.1–21.7)	19.4 (16–23.3)	<0.001
OI (*n* = 119)	39.6 (29.4–47.8)	22.6 (14.5–30.2)	33.3 (20.6–42.1)	<0.001
VIS	20 (10.5–41.9)	15 (5–30)	20 (10–40)	0.032
Values at 4 h after PICU arrival (Time B)				
A-a gradient (*n* = 123)	605.8 (587–625.5)	510.5 (435.1–582.6)	591.5 (525.8–617)	<0.001
SF ratio	88.5 (81–95)	99 (95–110.6)	93 (84–99)	<0.001
PF ratio (*n* = 123)	45 (31–63)	88.5 (61.5–164.4)	56 (38–86)	<0.001
OSI	20.9 (18.3–24.6)	16.2 (13.5–19.1)	19.4 (15.6–23.1)	<0.001
OI (*n* = 123)	40 (27.2–59.5)	18.2 (10.7–23.9)	30 (18.4–51.5)	<0.001
VIS	24.3 (15–24.3)	17 (7.5–29)	20 (13–37)	0.002

Abbreviations: A-a gradient—alveolar–arterial oxygen gradient; ECMO—extracorporeal membrane oxygenation; FiO_2_ = fraction of inspired oxygen; MAP—mean airway pressure; OI—oxygenation index; OSI—oxygen saturation index; PF ratio—ratio between paO_2_ and FiO_2_; SF—ratio between SpO_2_ and FiO_2_; VIS—vasoactive–inotropic score.

**Table 3:** Logistic regression analysis for variables that predict ECMO support.

	At PICU Arrival (Time-A)			At 4 h After PICU Arrival (Time-B)		
Variable	Beta-Value	Adjusted Odds Ratio (95% CI)	*p* Value	Beta-Value	Adjusted Odds Ratio (95% CI)	*p* Value
PHT	0.978	2.659 (1.23–5.746)	0.016	0.845	2.329 (1.052–5.156)	0.037
VIS	0.004	1.004 (0.991–1.016)	0.553	0.015	1.015 (0.998–1.033)	0.090
OSI	0.071	1.073 (1.002–1.149)	0.048	0.087	1.091 (1.023–1.164)	0.008
X2 (df)	13.495 (3)			22.854 (3)		
Model *p*-value	0.004			<0.001		
Nagelkerke R2	0.121			0.199		
H-L *p*-value	0.580			0.594		
C-static	0.682 (0.589–0.774)			0.780 (0.698–0.862)		

Abbreviations: ECMO—extracorporeal membrane oxygenation; H-L—Hosmer and Lemeshow test; OSI—oxygen saturation index; PHT—systemic or suprasystemic pulmonary pressures on echocardiogram; VIS—vasoactive–inotropic score.

## Data Availability

Data are not publicly available due to institutional restrictions. Data may be provided on request.

## Abbreviations

The following abbreviations are used in this manuscript:

OI	Oxygenation index
OSI	Oxygen saturation index
MAP	Mean airway pressure
FIO_2_	Fraction of inspired oxygen
MAS	Meconium aspiration syndrome
pHTN	Pulmonary hypertension
PPHN	Persistent pulmonary hypertension of the newborn
A-a gradient	Alveolar–arterial oxygen gradient
PF ratio	PaO_2_/FiO_2_ ratio
SF ratio	SpO_2_/FiO_2_ ratio
HFOV	High-frequency oscillatory ventilation
ECMO	Extracorporeal membrane oxygenation
ROM	Rupture of membranes
